# Mice Dually Disrupted for *Nod2* and *Mincle* Manifest Early Bacteriological Control but Late Susceptibility During *Mycobacterium tuberculosis* Infection

**DOI:** 10.3389/fimmu.2022.862992

**Published:** 2022-03-28

**Authors:** Jean-Yves Dubé, Fiona McIntosh, Marcel A. Behr

**Affiliations:** ^1^ Department of Microbiology and Immunology, McGill University, Montréal, QC, Canada; ^2^ Infectious Diseases and Immunity in Global Health Program, Research Institute of the McGill University Health Centre, Montréal, QC, Canada; ^3^ McGill International TB Centre, Montréal, QC, Canada; ^4^ Department of Medicine, McGill University Health Centre, Montréal, QC, Canada

**Keywords:** *Mycobacterium tuberculosis*, NOD2, Mincle, innate immunity, pattern recognition receptors

## Abstract

Pattern recognition receptors Mincle and NOD2 have been implicated in mycobacterial immunity. However, knockout (KO) animal infection studies with *Mycobacterium tuberculosis* (*Mtb*) have had mild/delayed phenotypes. Given that genetic susceptibility to infectious diseases can be polygenic, we hypothesized that murine double knockout (DKO) of *Mincle* and *Nod2* would result in exacerbation of altered immunity to mycobacterial infection leading to a more extreme phenotype than either KO alone. To test this hypothesis, we monitored bacterial burden, immune responses and survival following *in vivo* infections with *Mtb* in DKO mice for comparison to wildtype (WT) and single KOs. Bacterial burden and immune responses were not significantly affected at 3 and 6 weeks after infection in all mutant mice. At later timepoints, *Nod2*-KO mice had reduced survival compared to wildtype mice, and *Mincle*-KO survival was intermediate. Unexpectedly, dual disruption had no further effect; rather, DKO mice phenocopied *Nod2*-KO mice. We observed that *Mtb*-related death, exclusively in mice with disrupted *Nod2*, was accompanied by greater pulmonary cell death and distinct large necrotic foci. Therefore, determining how these receptors contribute to mycobacterial resistance will require analysis of immunophenotypes and their consequences on host pathology.

## Introduction

Each year, *Mycobacterium tuberculosis* (*Mtb*) causes tuberculosis (TB) in about 10 million people and results in the death of over a million (WHO). Other species in the *Mtb* complex as well as non-tuberculous mycobacteria cause disease in livestock and occasionally in humans too. Many aspects of immunity have been linked to susceptibility to mycobacterial infection (e.g. HIV status, host genetics). Understanding which immune mechanisms are important for host control or tolerance of mycobacteria is needed to guide the development of immunotherapeutic agents and vaccines.

Pattern recognition receptors (PRRs) help to initiate innate immunity and instruct the development of adaptive immunity to pathogens. They are a collection of genetically inborn sensors that detect microbe-associated molecular patterns (MAMPs) to initiate the inflammatory response at the cellular level (e.g. through NF-κB and MAPK pathways). One such PRR is Macrophage-Inducible C-type Lectin (Mincle), which binds to the mycobacterial outer membrane lipid trehalose 6,6’-dimycolate (TDM, aka cord factor) (1). TDM is an abundant glycolipid in the outer membrane of mycobacteria and has been posited as both a host-beneficial MAMP as well as a virulence factor of pathogenic mycobacteria (2). *Mincle*-/- mice have altered immunity to mycobacteria (1, 3–5), although whether *Mincle* polymorphisms are associated with human mycobacterial infection is only starting to emerge: at least two such studies reported statistically significant associations (6, 7).

Another PRR of interest is Nucleotide-binding Oligomerization Domain-containing 2 (NOD2), which is a cytoplasmic molecule essential for the recognition of the bacterial peptidoglycan through the muropeptide moiety (8–10). NOD2 has been implicated in mycobacterial immunity through chemical, immunologic and epidemiologic evidence: 1) mycobacteria contain a distinct NOD2 agonist in that they possess *N*-glycolylated muropeptides while other bacteria have *N*-acetylated muropeptides (11, 12); 2) *N*-glycolylation of the muropeptide was shown to be a more potent activator of innate immune cells (13, 14) and *Nod2*-/- mice have decreased immune responses to mycobacteria (15, 16); 3) polymorphisms in NOD2 have been associated with several mycobacterial diseases including tuberculosis, leprosy and Buruli ulcer (17–20).

We have recently shown that both *Mincle* and *Nod2* are essential to the immune response elicited by Freund’s adjuvant, a mycobacterial adjuvant, and that Mincle and NOD2 ligands together account for up to one half of the cellular immune response to dead *Mtb* (14). However, *Nod2*-/- mouse studies have shown only modestly reduced *Mtb* control (15, 16) and survival (16). *Mincle*-/- mice were shown to have little difference from WT in terms of *Mtb* burden and survival analysis has not been reported (4, 5). Because Mincle and NOD2 are relatively distinct PRRs which have the potential to work synergistically (13, 14), we hypothesized that simultaneous loss of *Mincle* and *Nod2* could result in a greater degree of altered immunity leading to a larger impairment in bacterial control and survival compared to single knockouts. The ‘double-knockout’ simulates a genetic ‘two-hit’ scenario which could explain why some loss-of-function polymorphisms do not appear to have complete disease phenotype penetrance. To address this, we used *Mincle*-/-*Nod2*-/- (double knockout, DKO) mice previously generated (14) (with the corresponding single knockouts, SKOs) to model genetic susceptibility to mycobacterial disease during infection with *Mtb*.

## Materials and Methods

### Mice

*Nod2*-/- mice were initially obtained from Jackson laboratories. *Mincle*-/- mice breeders were generously provided by the laboratory of Christine Wells (21). Imported mice were genotyped to confirm that the *Dock2* mutation reported in certain *Nod2*-/- mice was not present (22). All mice were obtained by crossing *Nod2*-/- and *Mincle*-/- mice (both C57BL/6 background) to generate double heterozygotes which were mated to regenerate single *Nod2* and *Mincle* knockouts as well as ‘wild-type’ (WT, *Mincle*+/+*Nod2*+/+) and ‘double knockout’ (DKO, *Mincle*-/-*Nod2*-/-). Homozygotic mice were then used as breeders to sustain the colony. All mice were from 2 to 4 months of age at the start of the experiment. Mice were age and sex matched where applicable across groups in all experiments. All protocols requiring mice adhered to the guidelines of the Canadian Council on Animal Care (CCAC) and were authorized by the RI-MUHC animal resource division.

### Bacteria

*Mtb* H37Rv was grown at 37°C in complete 7H9 medium [Middlebrook 7H9 (BD), ADC (BD), 0.2% glycerol (Sigma), 0.05% Tween-80 (Sigma)]. *Mtb* culture used in aerosol experiments was aliquoted into 7H9 complete medium with 20% glycerol at OD-600 ~ 0.5, frozen at -80°C and thawed immediately before use. For plating on solid media, we used complete 7H10 [Middlebrook 7H10 (BD), OADC (BD), 0.5% glycerol (Sigma)] including PANTA (BD).

### *Mtb* Aerosol Infections

Mice were infected by aerosol using an ONARES device (New Jersey, USA) over 15 minutes with a *Mtb* suspension in PBS + 0.05% Tween-80 at OD-600 = 0.04. This method routinely resulted in a low dose *Mtb* infection (< 60 CFU total in both lungs one day after infection). Actual day 1 pulmonary loads per experiment are indicated in figure legends.

### *Mtb* Survival

Mice infected with *Mtb* were weighed and periodically examined every 1-4 weeks with a body score system (4 = normal; 3 = slight hunch; 2 = hunch plus less active or reduced grooming; 2- = 2 with slow gait and very bad posture; 1 = hardly moving, dehydrated, thin and ungroomed). Endpoints were set to determine whether mice were imminently dying of *Mtb* infection and/or required compassionate euthanasia: body score under 2; body score of 2 with relatively low and rapidly dropping weight (such that death is likely within a week); body score of 3 with extremely low (<20 g) and steadily dropping weight (losing >1g/week for last 2 weeks); non-TB ailment normally requiring compassionate euthanasia (e.g. serious wound).

### Enumeration of Organ CFUs

Lungs, spleens and livers were removed from mice after euthanasia and processed immediately. Organs were placed in 1 ml complete 7H9 and were homogenized with an Omni Tissue Homogenizer TH (Omni International) for 30 seconds. Homogenate was serially diluted and plated. After approximately 3 weeks at 37°C, *Mtb* CFUs were enumerated from culture plates.

### Lung Cell Preparation for Flow Cytometry

Three minutes before euthanasia by cervical dislocation, anesthetized mice were injected intravenously with 5 μg of anti-CD45-FITC (BD) antibody to stain circulatory leukocytes for later differentiation from parenchymal leukocytes (see **Figure S1** for gating). Right lungs were harvested, sliced into small pieces in RPMI-1640 medium and digested in 150 U/ml collagenase (purified from *Clostridium histolyticum*, Sigma) for 1 hour at 37°C. Digested lung tissue was pressed through a 100 μM cell strainer, treated with red blood cell lysis buffer (Roche), and resuspended in a small volume of RPMI-1640. 20% of this suspension was taken for flow cytometry staining.

### Flow Cytometry Analysis

Cells were washed in PBS then stained with LIVE/DEAD™ fixable violet dead cell stain (ThermoFisher Scientific). Next, cells were washed with FACS buffer (PBS, 0.5% BSA, 2 mM EDTA), Fc receptors were blocked with TruStain FcX (Biolegend), and then cells were incubated with antibody cocktail prepared in a 1:1 mix of FACS buffer and BD Horizon™ Brilliant Stain Buffer. Cells were washed in FACS buffer, fixed in BD fixation/permeabilization solution, washed in FACS buffer and stored at 4°C until analysis. Equal numbers of Precision Count Beads™ (Biolegend) were added to samples just before analysis to determine total cell numbers per organ. A BD Fortessa X-20 cytometer was used for all experiments. Flow cytometry data were collected with FACSDivaTM software (BD) and FCS files were analyzed using FlowJo V10 (BD). Antibody probes from BD included (target, clone, fluorochrome): B220, RA3-6B2, BUV737; CD3ϵ, 145-2C11, PE; CD8α, 53-6.7, BV711; CD11c, HL3, BUV395; CD45, 30-F11, FITC; Siglec-F, E50-2440, BV786. Antibody probes from Biolegend included (target, clone, fluorochrome): CD4, GK1.5, BV510; CD11b, M1/70, PE-Cy7; CD19, 6D5, PE-Dazzle594; Ly6C, HK1.4, APC-Cy7; Ly6G, 1A8, PerCP-Cy5.5; MHC-II (I-A^b^), AF6-120.1, APC.

### Histology

For short-term (3-6 week) timepoints, the accessory (post-caval) lobe of the right lung was harvested and placed immediately in 10% formalin for storage until H&E processing. For survival experiments, after CO_2_ asphyxiation lungs were injected with 10% formalin through an intratracheal catheter *in situ* as described elsewhere (23). Briefly, a reservoir of formalin was maintained 25 cm above the mouse to provide a standardized pressure to the injection. A fluid line from this reservoir was fitted with an intravenous catheter which was inserted into an incision made in the trachea. Silk thread was tied around the trachea to seal the catheter in place, after which the stopcock was opened to allow formalin into the lungs at constant pressure which was maintained for about 20 minutes. After *in situ* fixation, lungs were stored in 10% formalin until H&E processing. All tissue processing post-fixation (embedding, cutting and staining) was performed by the Histology Core at the Goodman Cancer Research Centre of McGill University.

Digital images of H&E- and Ziehl–Neelsen-stained sections were generated with a Nikon Eclipse microscope or a Leica Aperio AT Turbo digital pathology scanner at 40X. For 3-6 week timepoints, one representative image of each accessory lobe was obtained for blinded ranking. For survival experiments, digitally scanned slides containing whole lung sections were examined and analyzed with QuPath (24) to quantify cell death by manually annotating lesions with cell death to calculate its area relative to the total lung area analyzed.

### Data Management and Statistical Analyses

Numerical data were collected with Microsoft Excel. All graphs were generated with GraphPad Prism v9 (GraphPad Software Inc.). Determinations of data normalcy and statistical significance were performed with the assistance of GraphPad. Figures were completed in Microsoft PowerPoint. The manuscript was written with Microsoft Word.

## Results

### *Mincle*-*Nod2* DKO Did Not Result in an Early Phenotype of Significant Impairment of *Mtb* Control

**Figure 1:**
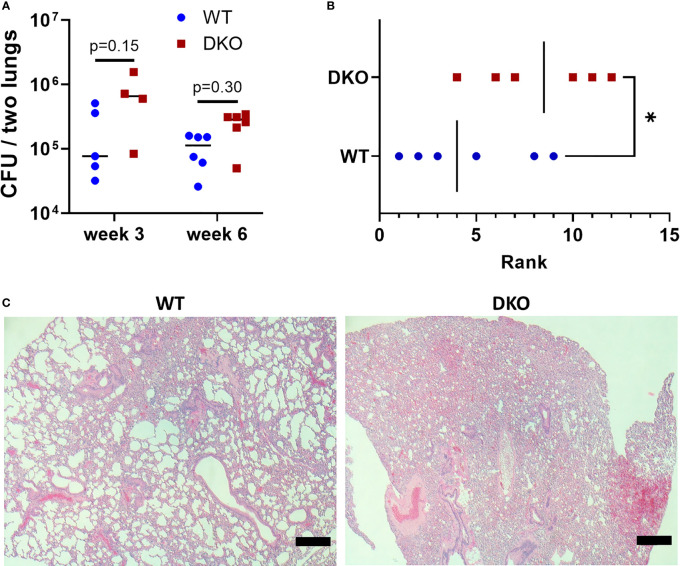
Effect of simultaneous *Mincle* and *Nod2* disruption on the pulmonary immune response to *Mtb*. **(A)**, total *Mtb* burden in both lungs of WT and DKO mice at week 3 (day 26) and week 6 (day 42) post-infection. No significant differences (α = 0.05) in *Mtb* burden were found using Dunn’s multiple comparisons test (p values shown on graph). **(B)**, rank of pathology on H&E lung sections at 42 days post-infection. Ranking was obtained from the median rank of seven blinded individuals. *p = 0.0465 with one-tailed Mann-Whitney test. **(C)**, representative H&E sections from WT (rank 5) and DKO (rank 10) mice. Scale bar, 200 μm. Median day 1 pulmonary *Mtb* load was 9 CFU. N = 4 to 6 mice per group.

We first sought to determine whether the dual absence of *Mincle* and *Nod2* resulted in an extreme phenotype of loss of *Mtb* control and/or severe immunopathology. At 3- and 6-weeks post-infection, the bacterial burden in DKO mice was not significantly increased compared to WT, although it tended to be greater (**Figure 1A**). At 6 weeks post-infection, blinded ranking of H&E-stained sections of lungs showed that DKO mice ranked with higher pathology (**Figure 1B**). DKO mice tended to have less alveolar airspace than WT (**Figure 1C**). We did not observe lymphocytic foci in DKO mice (**Figure S2**). Previous studies had shown *Mincle* and *Nod2* SKOs had reduced lymphocyte immunity and/or similarly small increases in bacterial burden compared to WT during mycobacterial infection (3, 5, 16). This result with DKO mice was therefore consistent with other SKO studies, showing an altered state of immunity early after infection.

### Survival of *Mtb*-Infected DKO Mice Was Decreased

**Figure 2:**
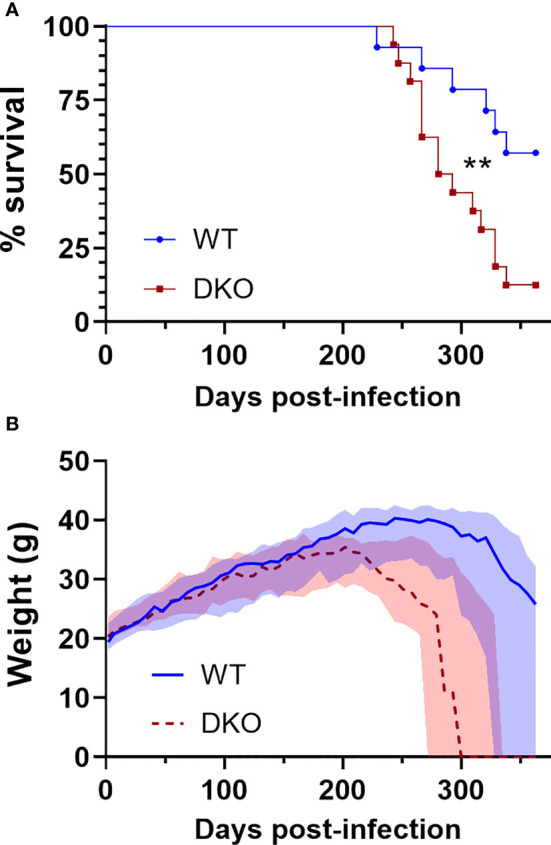
Survival of DKO mice infected with *Mtb* in comparison to WT. **(A)**, percent survival. **p = 0.0076 by log-rank (Mantel-Cox) test. **(B)**, weight in grams; median (lines) +/- interquartile range (shading) are shown. Euthanized mice received a weight of 0 g. All mice were female. Median day 1 pulmonary *Mtb* load was 29 CFU. N = 14 and 16 for WT and DKO, respectively.

To determine whether dual absence of *Mincle* and *Nod2* would result in impaired survival with *Mtb* infection, we infected WT and DKO mice with *Mtb* by aerosol and monitored them over the course of a year. Mice were euthanized when they reached a predefined endpoint (see *Methods*). The median survival of DKO mice was 287 days, while over half the WT mice were surviving by day 363, when the experiment was ended (**Figure 2A**). This difference in survival was statistically significant. The weight of the mice was measured throughout the experiment, and mice dying of *Mtb* infection lost much of their body mass in the weeks prior to death (**Figure 2B**). Thus, DKO mice were more susceptible to *Mtb* than WT mice, albeit at a late timepoint.

### Impact of DKO During Early *Mtb* Infection Was Similar to SKOs in Terms of Bacterial Burden and the Pulmonary Immune Response

**Figure 3:**
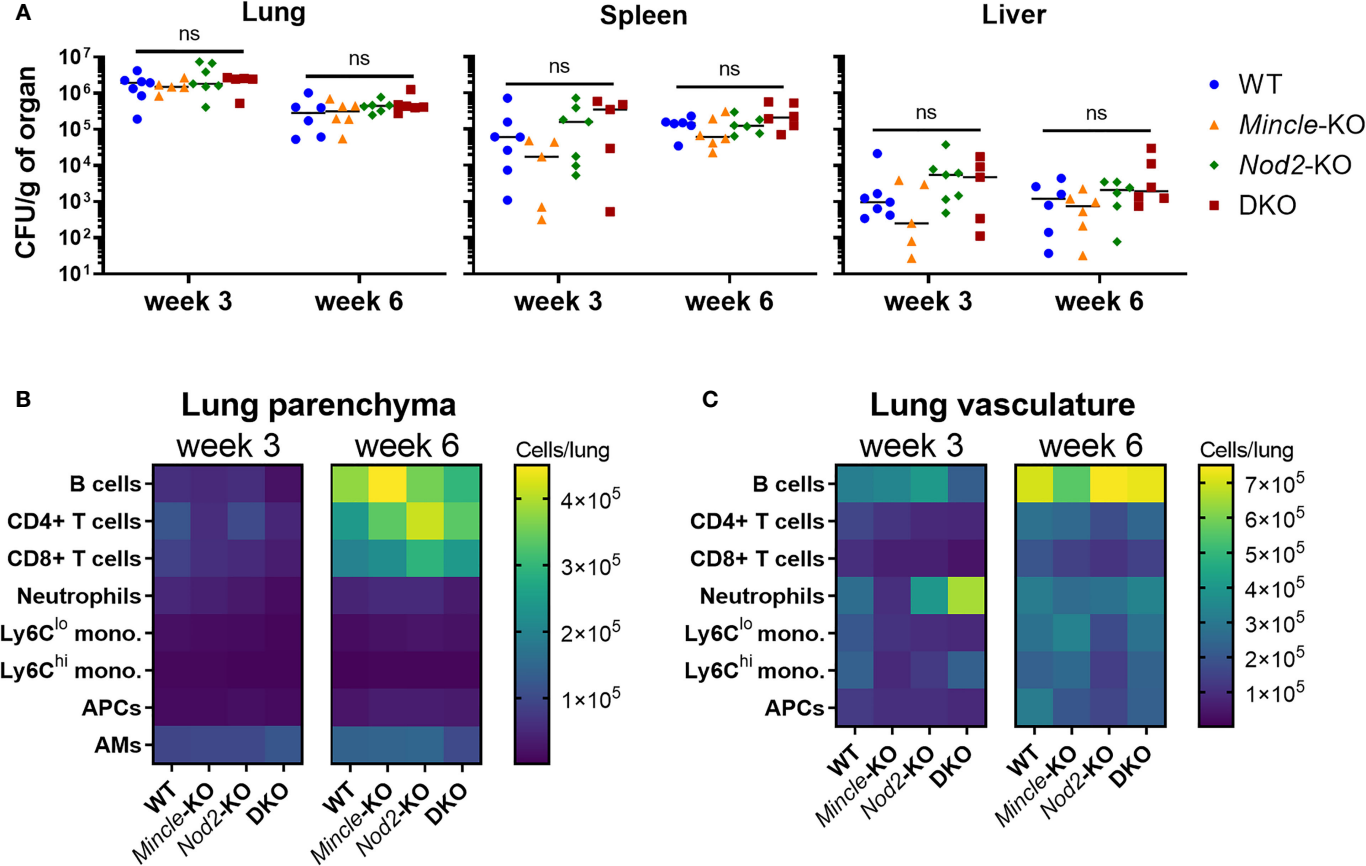
*Mtb* burden and leukocyte recruitment in mice lacking *Mincle* and *Nod2* singly and in combination. **(A)**, *Mtb* burden per gram of left lung (left), spleen (middle) and liver (right) at week 3 (day 22) and week 6 (day 43) post-infection. No statistically significant differences (α = 0.05) were found between genotypes using Kruskal-Wallis tests (ns, not significant). **(B, C)**, absolute numbers of pulmonary leukocytes at the indicated timepoints post-infection. Using intravenous anti-CD45-FITC staining just prior to euthanasia, parenchymal (CD45-FITC^-^) **(B)** or vascular (CD45-FITC^+^) **(C)** location was determined. See **Figure S3** for statistical comparisons of populations. Median day 1 pulmonary *Mtb* load was 15 CFU. Shown are medians for N = 5 to 7 mice per group per timepoint.

Given that late control of pulmonary *Mtb* infection was impaired in the absence of both *Mincle* and *Nod2* compared to WT, we sought to directly compare WT, SKOs and DKO mice during *Mtb* infection in terms of *Mtb* control and adaptive immune responses. We first set out to verify that there was little effect of each PRR on *Mtb* control and immune responses at the early timepoints. No statistically significant differences in *Mtb* burden between genotypes were found in lungs, spleens nor livers at 3 and 6 weeks (**Figure 3A**). Likewise, at 3 and 6 weeks post-infection, there were no significant differences in leukocyte numbers in the lung parenchyma (**Figure 3B**) nor vasculature (**Figure 3C**) (statistics in **Figure S3**). Any trends of impaired immune responses in KO lungs at 3 weeks (e.g. numbers of monocytes, neutrophils and lymphocytes) had disappeared by 6 weeks. Thus, despite previous reports of impaired immunity in *Nod2*-KO mice infected with *Mtb*, mostly gathered within two months post-infection (15, 16), our data show that any impairment in early cell recruitment tends to recover by 6 weeks post-infection. Importantly, DKO mice did not differ largely from SKO mice, contrary to our hypothesis.

### *Nod2* Deficiency Drove Decreased Survival in *Mtb*-Infected Mice Irrespective of *Mincle* Status

**Figure 4:**
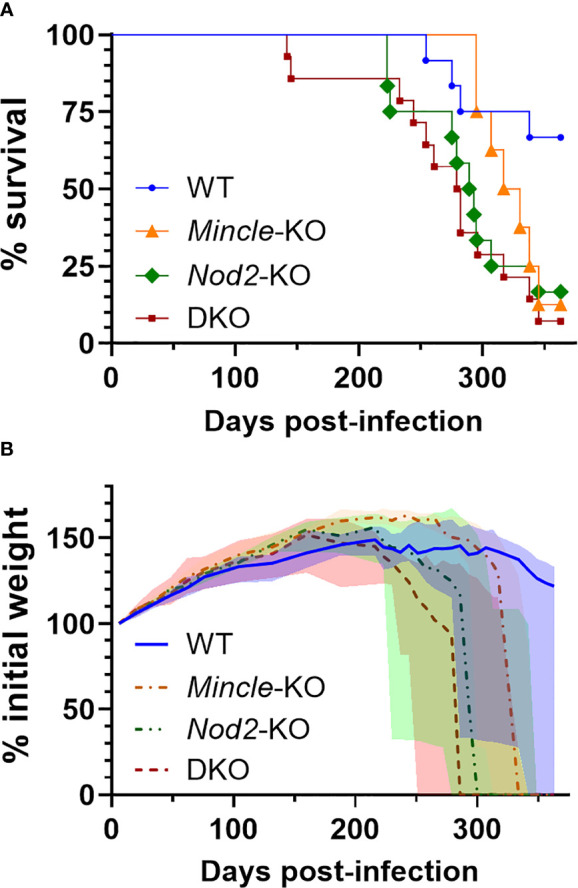
Survival of mice lacking *Mincle* and *Nod2* singly and in combination after infection with *Mtb*. **(A)**, percent survival. Log-rank (Mantel-Cox) test p values with Bonferrroni-correction for five comparisons, comparing to WT, equal 0.249, 0.0805 and 0.0080 for *Mincle*-KO, *Nod2*-KO and DKO, respectively; for comparison to DKO, corrected p values equal 0.202 and > 0.99 for *Mincle*-KO and *Nod2*-KO, respectively. **(B)**, percent weight from 6 days post-infection over time for the indicated genotype (male and female mice combined); median (lines) +/- interquartile range (shading) are shown. Euthanized mice received a weight of 0. Weight in grams is plotted in **Figure S4**. Median day 1 pulmonary *Mtb* load was 31 CFU. N = 12, 8, 12 and 14 for WT, *Mincle*-KO, *Nod2*-KO and DKO, respectively.

*Nod2*-KO mice had been shown to have reduced survival during *Mtb* infection compared to WT mice in one previous study (16). We could not find any published work presenting a survival curve for *Mtb* infection in *Mincle*-KO mice. In a direct comparison of DKO mice to WT mice, we observed earlier mortality in the DKO mice. To determine whether this decrease in survival was attributable to *Nod2* and/or Mincle, we aerosol infected DKO and SKO mice in parallel with WT mice. Median survival times of *Mincle*-KO, *Nod2*-KO and DKO groups were 324, 291 and 281 days, respectively (**Figure 4A**). The experiment was ended at day 363 with more than half of WT mice surviving (**Figure 4A**). Compared to WT, the probabilities (uncorrected/Bonferroni-corrected) that SKO and DKO groups had significantly reduced survival were p = 0.0497/0.249, 0.0161/0.0805 and 0.0016/0.0080 for *Mincle*-KO, *Nod2*-KO and DKO, respectively; compared to DKO, neither *Mincle*-KO nor *Nod2*-KO survival were significantly different. *Nod2*-KO and DKO curves closely followed each other; the *Mincle*-KO curve landed in between curves for WT and *Nod2*-KO mice. Mice dying of *Mtb* infection were losing weight prior to reaching the euthanasia endpoint (**Figure 4B**). Mass of male and female mice are plotted separately in **Figure S4**. By weight, DKO and *Nod2*-KO groups were ill earlier than *Mincle*-KO and WT groups.

### Absence of *Nod2* Was Associated With Altered Immunity Near *Mtb*-Related Mortality

**Figure 5:**
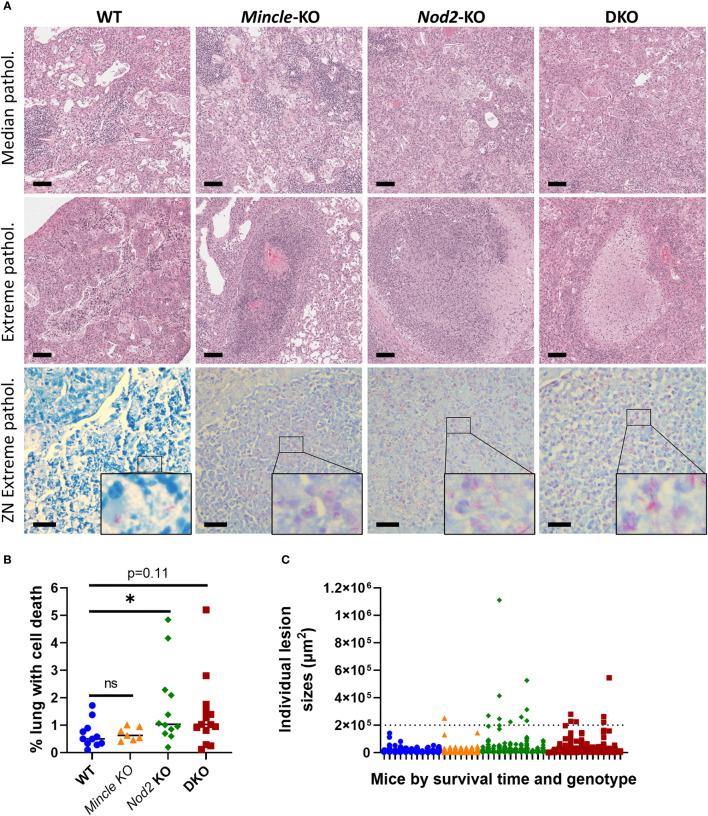
Pulmonary pathology at death in *Mincle* and *Nod2* deficient mice infected with *Mtb*. **(A)**, representative images of H&E-stained sections showing median pathology by genotype (top row), largest lesions containing cell death by genotype (middle row, center of images, including necrotizing foci for *Nod2*-deficient animals) and ZN-stained sections corresponding to lesions in middle (bottom row, with enlarged inset to show AFB). Scale bars for H&E and ZN are 100 μm and 20 μm, respectively. **(B)**, percentage of lung space containing dead cells with medians. Groups were significantly different by a Kruskal-Wallis test: p = 0.0424. Shown in the panel are the results of Dunn’s multiple comparisons test for comparison of *Mincle*-KO, *Nod2*-KO and DKO to WT; ns, not significant (p > 0.05); *p = 0.0486. **(C)** sizes of individual lesions containing dead cells, separated by mouse according to genotype (colour/shape-coded) and in order of survival time (left to right, shortest to longest). Dotted line delineates lesions larger than 0.2 mm^2^. N = 11, 7, 12 and 14 for WT, *Mincle*-KO, *Nod2*-KO and DKO, respectively.

From mice in survival experiments, lungs were taken at euthanasia with formalin fixation *in situ* and were subsequently sectioned and stained with H&E and the ZN method. This was done to explore whether the pulmonary pathology associated with death from *Mtb* infection differed across the genotypes. Representative images of median pathology per genotype are shown in **Figure 5A**, top row. Across all four genotypes, we observed some alveoli free of inflammation as well as other sites of acute and chronic inflammation. We also observed lymphocytic foci, cell death, cholesterol accumulation and keratinization in all four groups. Interestingly, we only observed large, necrotizing foci in *Nod2*-KO and DKO lungs although these were in a minority of mice (3 of 12 *Nod2*-KO mice, and 1 of 14 DKO mice). These necrotizing foci where about half to one millimetre in diameter, circumscribed with a cuff of foamy macrophages and having a center containing cell debris mixed with few relatively intact foamy macrophages, PMNs and lymphocytes. The largest lesions containing cell death per genotype, including the necrotizing foci, are presented in **Figure 5A**, middle row. The aforementioned necrotizing foci were not seen in WT nor *Mincle*-KO mice. The largest lesions in WT were diffuse, lacking shape and contained less dead cells. In *Mincle*-KO mice, the largest lesions had less cell death and were bounded by epithelial cells of a relatively intact airway rather than foamy macrophages (**Figure 5A**, middle row and **Figure S5A**). To determine if these extreme lesions were associated with loss of bacterial control, we examined corresponding ZN-stained adjacent sections. Large lesions in SKOs and DKO mice were associated with an excess of acid-fast bacilli, while large lesions in WT mice were not (**Figure 5A**, bottom row).

Upon discovering these large necrotic foci containing uncontrolled bacteria, we sought to measure whether there was more cell death overall in the lungs of mice deficient in *Nod2*. The numbers of lesions containing dead cells were not significantly different between genotypes (**Figure S5B**). However, we observed a trend to a larger proportion of the lung volume containing dead cells in *Nod2*-KO and DKO mice compared to WT and *Mincle*-KO mice, which reached statistical significance for *Nod2*-KO mice alone (**Figure 5B**). This did not appear to be driven by the timing of euthanasia (**Figure S5C**). There were more large lesions (>0.2 mm^2^) containing cell death in *Nod2*-deficient mice compared to *Nod2*-competent mice (**Figure 5C**) which correlates with greater cell death overall and is compatible with the unique generation of large necrotic foci.

## Discussion

The PRRs Mincle and NOD2 uniquely interact with mycobacteria and have been shown to synergistically drive immunological functions (1, 13, 14). In this study, we used measures of bacterial control, the immune response and survival to examine the course of *Mtb* infection in mice defective for Mincle and/or NOD2. Consistent with other papers, these PRRs did not contribute alone to mycobacterial control early in infection; when disrupted together, there was no evidence of an added effect. Both PRRs contributed alone to prevent premature death in *Mtb*-infected mice, however. *Nod2*-KO and DKO mice had very similar reduced survival times, refuting the hypothesis that the double mutant would manifest severe susceptibility to *Mtb* infection. Our data indicate that PRRs like Mincle and NOD2 were dispensable for mounting immunity to and short-term control of *Mtb*, but that the absence of NOD2 eventually resulted in a novel and immunologically distinct death phenotype of increased pulmonary necrosis.

In humans, one study found a significant association between *NOD2* loss-of-function mutations and an increased risk of necrotizing enterocolitis or focal intestinal perforation in very low-birth-weight infants (25), suggesting NOD2 deficiency is related to necrotic pathology in humans. Murine *Nod2* and MDP treatment promoted the formation of necrotic zones in a murine model of atherosclerosis however (26), so the relationship between NOD2 and necrosis seems complex. The necrotic foci seen in *Nod2* deficient mice were reminiscent of similar pathology in the Kramnik mouse (27, 28), the susceptibility of which has been attributed to the *Sst1* locus and excess type I IFN production (29). However, NOD2 signaling contributed to type I IFN production (30), so how the absence of *Nod2* lead to the formation of necrotic foci is not so easily explainable. Perhaps the amount of type I IFN in space and time is a critical element for these outcomes, or that excess type I IFN precipitates a common secondary effect that leads to runaway necrosis. Heterozygous deficiency in IL-1R antagonist or anti-IL-1R antagonist treatment ameliorated morbidity and lung pathology in mice with the susceptible *Sst1* allele (29). IL-1 signaling contributed to PGE2 production, and PGE2 was protective against *Mtb* in IL-1 signaling-deficient mice (31). Thus, roles for NOD2 in IL-1 and PGE2 pathways should be further explored in late *Mtb* infection. When infected with *Mtb*, human PBMCs with functional NOD2 produced more IL-1 than human PBMCs containing the NOD2 3020insC mutation (32). MDP also promoted PGE2 production by monocytes and macrophages (33, 34). The balance of immune mediators like type I IFN, IL-1 and PGE2 may play a key role in pulmonary cell death during *Mtb* infection.

Extensive necrosis was not seen in *Nod2*-deficient mouse lungs at early timepoints (3 and 6 weeks post-infection) (**Figure 1C** and not shown). The phenotype of large necrotic foci was late, appearing in mice euthanized at 223, 275, 307 (*Nod2*-KO) and 254 (DKO) days post-infection, and only occurred in a minority of the animals. It will be challenging to directly study the mechanisms of this phenomenon with this model. A 10X higher-dose aerosol may increase the rate of death in *Nod2*-KO mice (16), but could overlook the phenotype that occurred with our more epidemiologically relevant dose of *Mtb* if time is needed for the pathology to develop. At 6 weeks post-infection, DKO mouse lungs were more congested than WT controls and but without lymphocytic clusters (**Figure 1C**), consistent with what has been demonstrated before with *Nod2*-KO mice (16). Our trend of decreased lymphocytes in *Nod2*-KO and DKO mouse lungs at three weeks post-infection with *Mtb* is similar to results with *Nod2*-KO mouse lungs at 4 weeks post-infection with BCG Russia (16). The defect in innate immunity caused by *Nod2* mutation results in altered instruction of the adaptive immune response (14, 16), but while the innate defect is undoubtedly present throughout the course of the infection (and therefore well deserves attention), we are only certain of an early adaptive defect. NOD2 deficiency may also impair macrophage production of nitric oxide as well as secreted immune mediators (15).

Throughout this study, what had not shone through is the importance of Mincle. *Mincle*-KO mice demonstrated a null or intermediate phenotype between WT and *Nod2-*deficient animals. Most noteworthy was the slight reduction in survival with *Mtb* for *Mincle*-KO mice compared to WT, which to our knowledge has not been published before. Previous *Mincle*-KO studies of *Mtb* infection did not present survival data but had only reported bacteriologic and immunologic changes which were inconsistent between studies (4, 5). Administration of TDM or synthetic TDM analogues has been shown to produce distinct, robust biologic effects (1, 14) but this is dependent on an arbitrarily selected dose which may not be representative of the amount of immune-available material from the millions of bacilli in a mouse. Interestingly, as *Nod2*-KO and DKO mice phenocopied each other in terms of survival with *Mtb*, NOD2 appears to be dominant over Mincle in this system. This result suggests Mincle is dispensable in the absence of NOD2 and/or NOD2 deficiency simply leads to death before Mincle deficiency has an effect.

NOD2 polymorphisms are strongly associated with Crohn’s disease yet a long-hypothesized role for mycobacteria in Crohn’s disease remains unproven. Associations of *NOD2* polymorphisms and TB have not been as strong, but future studies exploring how altered immunity in NOD2-deficient hosts leads to distinct disease states will inform the definitions of TB endophenotypes which can be tested for associations with *NOD2* polymorphisms at large. The importance of PRRs during *Mtb* infection may indeed be restricted to specific phenotypes due their nature as redundant sensors of infection, in contrast to the essentiality of unique immune effectors (35). Our work has asserted that NOD2 is important in *Mtb* infection through reduced survival associated with distinct pathology. Defining the mechanisms of these NOD2 phenotypes will inform our understanding of human mycobacterial diseases.

## Data Availability Statement

The original contributions presented in the study are included in the article/**Supplementary Material**. Further inquiries can be directed to the corresponding author.

## Ethics Statement

The animal study was reviewed and approved by The Facility Animal Care Committee of the Animal Resources Division of the Research Institute of the McGill University Health Centre.

## Author Contributions

Conceptualization, J-YD and MB. Methodology, J-YD and FM. Investigation, J-YD and FM. Resources, MB. Writing – original draft, J-YD and MB. Writing – review and editing, J-YD, FM, and MB. Visualization, J-YD. Supervision, MB. Funding acquisition, J-YD and MB. All authors contributed to the article and approved the submitted version.

## Funding

J-YD was personally supported by the Canadian Institutes of Health Research (CIHR) Canada Graduate Scholarship – Master’s Program, Fonds de Recherche du Québec – Santé (FRQ-S) Doctoral Training Award, RI-MUHC studentships (Master’s and Ph.D.) and scholarships from the McGill Department of Microbiology and Immunology. Research activities were funded by a CIHR foundation grant held by MB (FDN-148362).

## Conflict of Interest

The authors declare that the research was conducted in the absence of any commercial or financial relationships that could be construed as a potential conflict of interest.

## Publisher’s Note

All claims expressed in this article are solely those of the authors and do not necessarily represent those of their affiliated organizations, or those of the publisher, the editors and the reviewers. Any product that may be evaluated in this article, or claim that may be made by its manufacturer, is not guaranteed or endorsed by the publisher.
