# Supplementary material for: Mice Dually Disrupted for Nod2 and Mincle Manifest Early Bacteriological Control but Late Susceptibility During Mycobacterium tuberculosis Infection
Source: Front Immunol. 2022 Mar 28;13:862992. doi: 10.3389/fimmu.2022.862992 (PMC8995500; doi:10.3389/fimmu.2022.862992)
Supplement: Supplementary file 1 [file DataSheet_1.pdf]

Mice dually disrupted for *Nod2* and *Mincle* manifest early bacteriological control but late susceptibility during *Mycobacterium tuberculosis* infection

Jean-Yves Dubé [1, 2, 3], Fiona McIntosh [2, 3], Marcel A. Behr [1, 2, 3, 4*]

[1] Department of Microbiology and Immunology, McGill University, Montréal, Canada.

[2] Infectious diseases and Immunity in Global Health Program, Research Institute of the McGill University Health Centre, Montréal, Canada.

[3] McGill International TB Centre, Montréal, Canada.

[4] Department of Medicine, McGill University Health Centre, Montréal, Canada.

<u>Supplemental Data</u>

5 figures

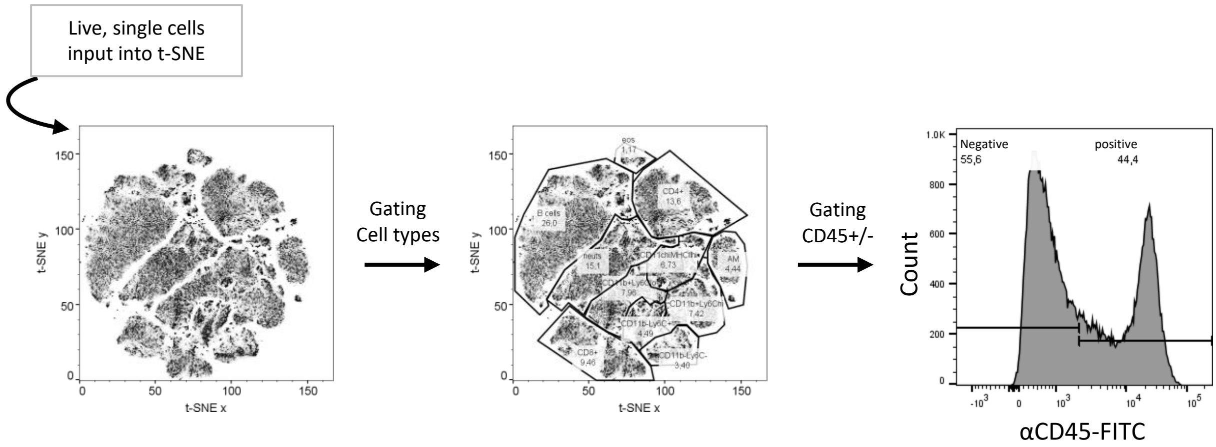

Figure S1. Flow cytometry gating strategy used to enumerate lung cell types. After gating on single, live cells, downsampling and concatenation were performed to put all samples through t-SNE. Populations in the two t-SNE-generated dimensions were identified and gated by checking expression of characteristic markers. Per population, gating of CD45-FITC positive and negative cells was performed separately to account for autofluorescence of difference leukocyte types. Total numbers of events in each final gate were exported and proportioned based on the number of counting beads passed with the cells to determine total cell type numbers per lung.

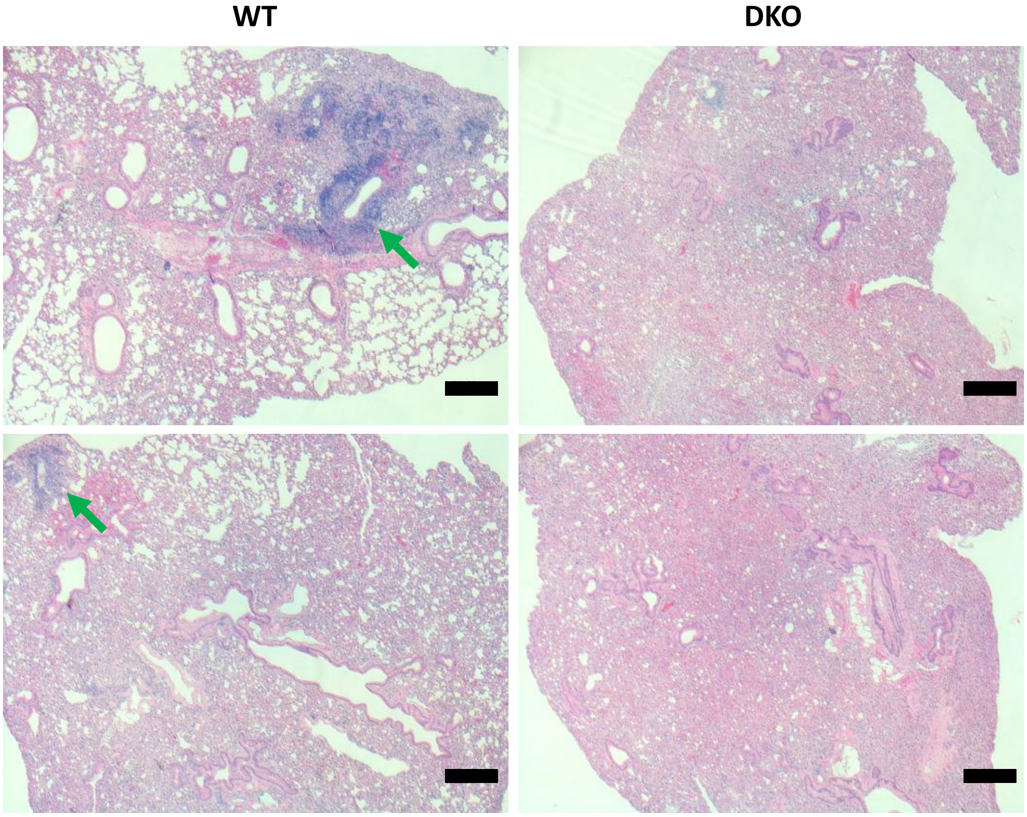

Figure S2. H&E-stained lung sections showing lymphocytic foci (green arrows) from WT mice (rank 8 and 9) , compared to DKO mice (rank 11 and 12), from experiment shown in fig. 1. Scale bar, 200 μm.

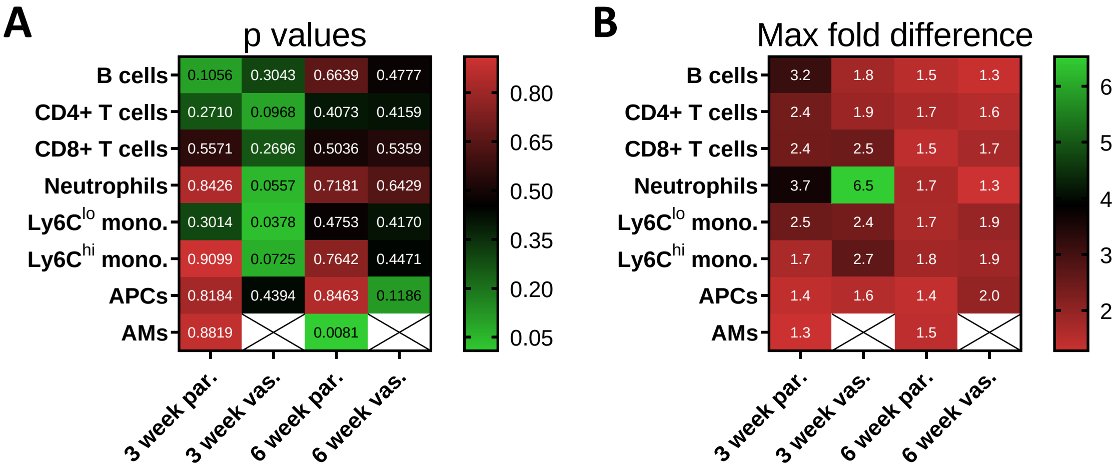

Figure S3. Statistics for lung flow cytometry presented in fig. 3B-C. **A**, p values of multiple Kruskal-Wallis tests performed across the four genotypes for the indicated populations and timepoints. No corrections for multiple comparisons were applied. **B**, fold difference between the lowest and highest genotype median for the indicated populations and timepoints (i.e. maximum median / minimum median). par., parenchymal-associated (CD45-) lung cells; vas., vasculature-associated (CD45+) lung cells.

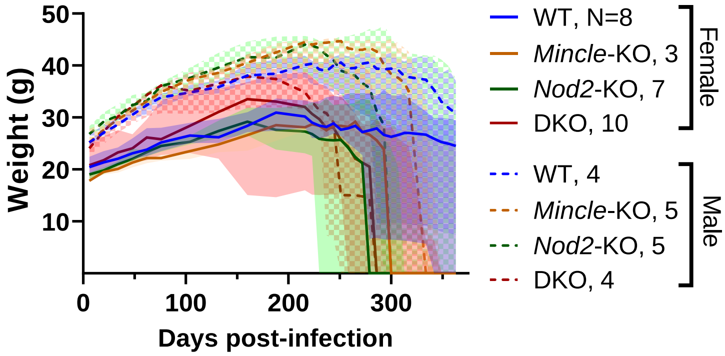

Figure S4. Weight of *Mincle* and *Nod2* deficient mice infected with *Mtb*. Weight in grams of mice subjected to survival challenge shown in fig. 4.

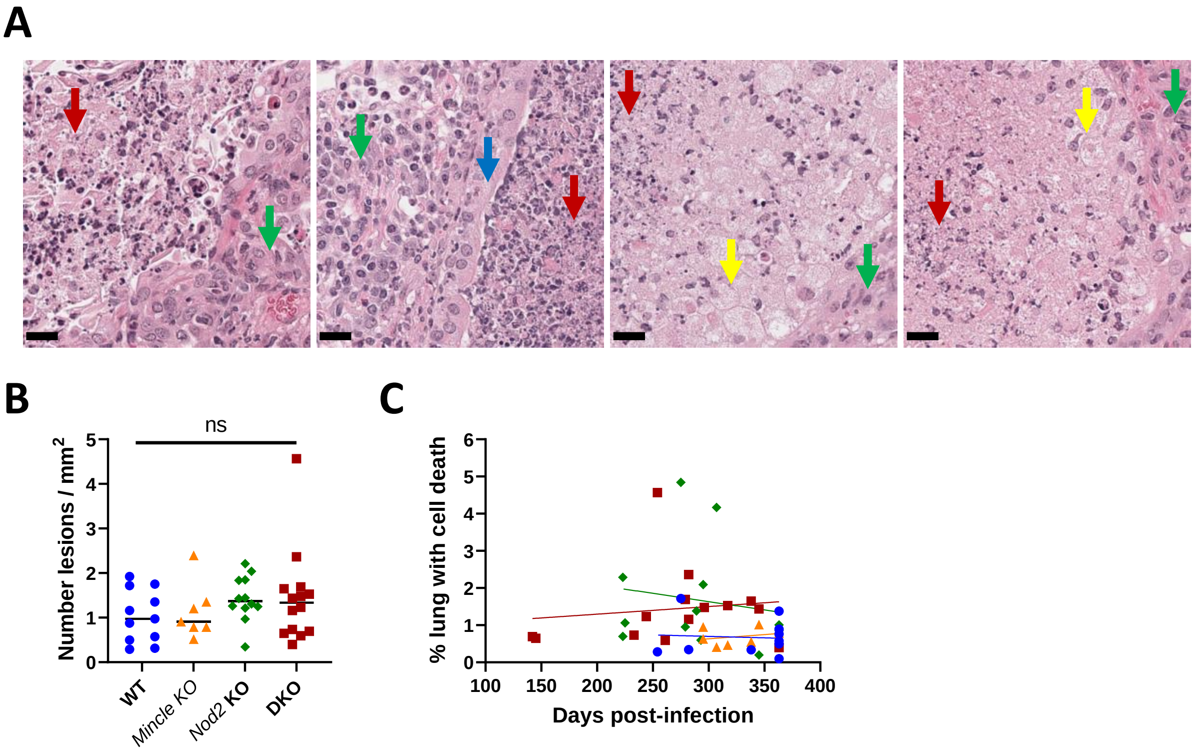

Figure S5. Quantitation of pulmonary pathology associated with death in *Mtb*-infected *Mincle* and *Nod2* deficient mice. **A**, Closeup of H&E sections showing extreme lung pathology with parts of interest indicated by arrows: green, inflamed tissue without cell death; red, region with cell death; blue, airway epithelial cells; yellow, foamy macrophages. Scale bar, 20 µm. **B**, Number of lesions containing dead cells per area of lung plotted with medians. Kruskal-Wallis test was used determine statistical significance; ns, $p > 0.05$. **C**, percentage of lung space containing dead cells plotted over time per group with linear regressions. No slopes were significantly non-zero.
